# Retrospective analysis of LNM risk factors and the effect of chemotherapy in early colorectal cancer: A Chinese multicenter study

**DOI:** 10.1186/s12885-020-07363-6

**Published:** 2020-11-05

**Authors:** Chunyan Zeng, Dandan Xiong, Fei Cheng, Qingtian Luo, Qiang Wang, Jun Huang, Guilian Lan, Huan Zhong, Youxiang Chen

**Affiliations:** 1 Department of Gastroenterology, the First Affiliated Hospital of Nanchang University, 17 Yongwaizheng Street, Nanchang, 330006 Jiangxi China; 2 Department of Gastroenterology, the Third Affiliated Hospital of Nanchang University, Nanchang, China; 3 Department of Gastroenterology, the Affiliated Ganzhou Hospital of Nanchang University, Ganzhou, China; 4 Department of Gastroenterology, Jiangxi Provincial People’s Hospital, Nanchang, China; 5 Department of Gastroenterology, Jiangxi Cancer Hospital, Nanchang, China; 6 Department of Biology, Hong Kong Baptist University, Hong Kong, China

**Keywords:** Early colorectal cancer, Chemotherapy, Lymph node metastasis, Risk factors, Overall survival, Recurrence

## Abstract

**Background:**

Estimating the risk of lymph node metastasis (LNM) is crucial for determining subsequent treatments following curative resection of early colorectal cancer (ECC). This multicenter study analyzed the risk factors of LNM and the effectiveness of postoperative chemotherapy in patients with ECC.

**Methods:**

We retrospectively analyzed the data of 473 patients with ECC who underwent general surgery in five hospitals between January 2007 and October 2018. The correlations between LNM and sex, age, tumor size, tumor location, endoscopic morphology, pathology, depth of invasion and tumor budding (TB) were directly estimated based on postoperative pathological analysis. We also observed the overall survival (OS) and recurrence in ECC patients with and without LNM after matching according to baseline measures.

**Results:**

In total, 473 ECC patients were observed, 288 patients were enrolled, and 17 patients had LNM (5.90%). The univariate analysis revealed that tumor size, pathology, and lymphovascular invasion were associated with LNM in ECC (*P* = 0.026, 0.000, and 0.000, respectively), and the multivariate logistic regression confirmed that tumor size, pathology, and lymphovascular invasion were risk factors for LNM (*P* = 0.021, 0.023, and 0.001, respectively). There were no significant differences in OS and recurrence between the ECC patients with and without LNM after matching based on baseline measures (*P* = 0.158 and 0.346, respectively), and no significant difference was observed between chemotherapy and no chemotherapy in ECC patients without LNM after surgery (*P* = 0.729 and 0.052).

**Conclusion:**

Tumor size, pathology, and lymphovascular invasion are risk factors for predicting LNM in ECC patients. Adjuvant chemotherapy could improve OS and recurrence in patients with LNM but not always in ECC patients without LNM.

## Background

Recently, as a result of the advocacy for endoscopic screening projects, the number of documented cases of early colorectal cancer (ECC) has increased [1]. ECC is defined as cancer located in the mucosa or submucosa with or without lymph node involvement (T1 TNM stage). Endoscopic treatment is absolutely the best choice of treatment for intramucosal ECC patients with no lymph node metastasis (LNM) and vascular invasion [2–4]. However, it has been reported that the LNM rate is as high as 7–15% in T1 colorectal cancer [5–8].

Therefore, endoscopic treatment can accomplish local primary tumor resection but not lymphadenectomy, and using this procedure for the radical excision of ECC with LNM undoubtedly must increase the postoperative recurrence rate and unfavorable prognosis. Moreover, preoperatively determining whether ECC is associated with lymph node metastasis is critical for selecting a surgical approach and the extent of resection. Although previous studies have reported that poor differentiation, the submucosal invasion depth, lymphovascular invasion, and tumor budding (TB) are risk factors for LNM, sufficient evidence suggesting that a particular risk factor affects long-term prognosis and the efficiency of postoperative chemotherapy is lacking. Thus, more evidence derived from long-term surveillance is needed. Furthermore, there is no consensus regarding the survival benefit of postoperative chemotherapy in early colon cancer [9].

In this study, our aim is to further analyze the risk factors in ECC patients with LNM in relation to various clinicopathologic characteristics. Moreover, we evaluate the effect of adjuvant chemotherapy following curative surgery.

## Methods

### Patient selection and data collection

**Fig. 1:**
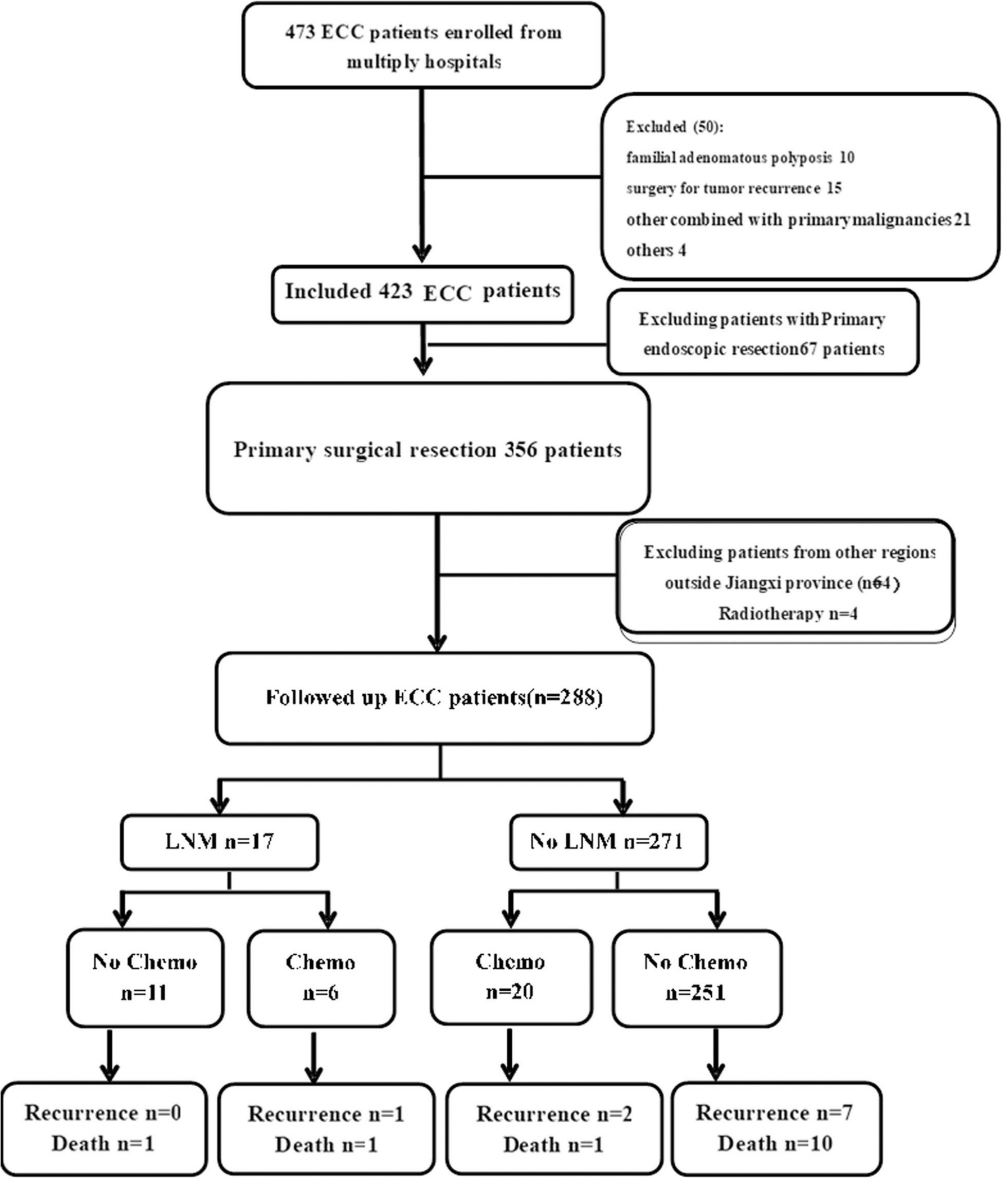
Flowchart of the enrolled patients. Abbreviations: ECC, early colorectal cancer; LNM, lymph node metastasis; Chemo, chemotherapy

The demographic and clinical data of 473 individuals who underwent endoscopic treatment and general surgery in our hospital and four other affiliated hospitals were retrospectively collected between January 2007 and August 2018. The inclusion criteria were as follows: all cases diagnosed with ECC by a postoperative pathological analysis. The exclusion criteria were as follows: recurrence after surgical resection, advanced colorectal cancer, presence of other primary malignant tumors, patients undergoing perioperative radiotherapy and preoperative chemotherapy, endoscopic resection of ECC and patients with familial adenomatous polyposis. Finally, all patients (288 patients after surgery) in the Jiangxi Province region were followed until November 30th, 2018 (Fig. 1). Moreover, the following data associated with chemotherapy were recorded: regimens, drugs, and times of treatment. The indications for chemotherapy were the presence of LNM or risk factors for LNM in ECC patients, such as poorly differentiated carcinomas, submucosal invasion, lymphovascular invasion, or TB. We established a collaborative study group involving five hospitals in two cities of Jiangxi Province, China. The study group confirmed that the design and data collection of this retrospective research were performed in accordance with the relevant guidelines and regulations. The clinical data was acquired with the approval and permission of the Institutional Review Board of the First Affiliated Hospital of Nanchang University.

### Clinicopathological parameters

The following demographic and clinicopathological data were recorded: sex, age, tumor size (in maximum diameter), tumor location, endoscopic morphological type, depth of invasion, pathology, lymphovascular invasion, TB and LNM. The tumor differentiation data are based on the 2010 WHO colorectal cancer pathology grading standard as follows: colorectal cancer is divided into highly differentiated, moderately differentiated, poorly differentiated, and undifferentiated carcinoma. According to the morphology of the tumor under endoscopy, ECC is divided into the uplift type (Ip, Isp, and Is), the flat type (IIa, IIb, IIa + dep, nongranular type LST, and granular LST), and the concave type (IIc, IIc + IIa, and Is+IIc).

### Statistical analysis

IBM SPSS statistics (version 20.0) was applied for the statistical analysis. A chi-squared test or t-test was used to analyze the relationship between the clinicopathological data and LNM in ECC. A logistic regression was used for the multivariate analysis of the factors identified as significant in the univariate analysis. A log-rank test was used in the tumor recurrence and overall survival analysis. *P* < 0.05 was considered indicative of statistical significance.

## Results

### Clinicopathological parameters (Table 1)

Two hundred and eighty-eight patients who underwent surgery with or without lymph node dissection were enrolled (male:female = 162:126). The age of the patients ranged from 27 to 82 years. The cancer was located in the rectum in 186 patients, sigmoid colon in 68 patients, ascending colon in 16 patients, descending colon in 10 cases, and transverse colon in 4 cases. Based on the endoscopic morphological classification, there were 248 cases of type I (including the uplift type, Ip, Isp, and Is), 7 cases of type II (including the flat type, IIa, IIb, IIa + dep, nongranular type LST, and granular LST), and 25 cases of type III (including the concave type, IIc, IIc + IIa, and Is+IIc). The diameter of the mass ranged from 7 to 120 mm (30.0 ± 15.6 mm). Regarding the pathological grading, 78 cases were highly differentiated, 202 cases were moderately differentiated, and 8 cases were poorly differentiated. As for the depth of invasion, 76 cases infiltrated the mucosal layer, while 212 cases infiltrated the submucosal layer. In total, 6 patients had lymphovascular invasion, and 3 cases had TB.

**Table 1:** Univariate analysis of risk factors and occurrence of lymph node metastasis

Factors	N	LNM(−),n(%)	LNM(+),n(%)	*P-value*
**N**	288	271	17	
**Gender**				0.431
Male	162	154 (53.47)	8 (47.06)	
Female	126	117 (46.53)	9 (52.94)	
**Age,χ ± SD (years old)**	59.5 + 11.6	59.7 + 11.7	56.8 + 11.2	0.316
**Smoking**				0.084
Positive	68	67 (23.26)	1 (5.88)	
Negative	220	204 (76.74)	16 (94.12)	
**Alcohol**				0.520
Positive	43	41 (15.13)	2 (11.76)	
Negative	245	230 (84.87)	15 (88.24)	
**Family history**				0.557
Positive	10	10 (3.69)	(0.00)	
Negative	278	261 (96.31)	17 (100.00)	
**Tumor size, χ ± SD (mm)**	30.0 ± 15.6	29.2 ± 15.4	38.3 ± 17.3	0.026*
**Tumor location**				0.115
Rectum	186	171 (63.10)	15 (88.24)	
Sigmoid colon	68	68 (25.09)	0	
Ascending colon	19	17 (6.27)	2 (11.76)	
Descending colon	10	10 (3.69)	0	
Transverse colon	5	5 (1.85)	0	
**Endoscopic morphology**				0.703
I (the uplift type)	248	232 (85.61)	16 (94.12)	
II (the flat type)	7	7 (2.58)	0 (0.00)	
III (the concave type)	25	24 (8.86)	1 (5.88)	
Uncertain	8	8 (2.95)	0 (0.00)	
**Pathology**				< 0.001**
Highly differentiated	78	78 (28.78)	0 (0.00)	
Moderately differentiated	202	189 (69.74)	13 (76.47)	
Poorly differentiated	8	4 (1.48)	4 (23.53)	
**Depth of invasion**				0.255
Mucosal layer	76	74 (27.31)	2 (11.76)	
Submucosal layer	212	197 (72.69)	15 (88.24)	
**Lympho-vascular invasion**				< 0.001**
Positive	6	2 (0.74)	4 (23.53)	
Negative	282	269 (99.26)	13 (76.47)	
**Tumor budding**				0.833
Positive	3	3 (1.11)	0 (0.00)	
Negative	285	268 (98.89)	17 (100.00)	

Note: * P < 0.05, ** P < 0.01. LNM(−):No lymph node metastasis, LNM(+):Lymph node metastasis

### Univariate analysis of the factors associated with LNM in ECC

LNM was more prevalent among the patients with a larger tumor size (*P* = 0.026 < 0.05). Furthermore, the rate of LNM in the uplift type was higher than that in the other endoscopic types in all patients. In the LNM group, there were 4 cases with the poorly differentiated type (23.53% vs. 1.48%, LNM vs. no-LNM group), and the other cases presented with a moderately differentiated type (76.47% vs. 69.74%, LNM vs. no-LNM group) (*P* < 0.01). Regarding the depth of invasion, there were no significant differences (*P* > 0.05) between the LNM and no-LNM groups (88.24% vs. 72.69%). Furthermore, the rate of lymphovascular invasion in the cases with LNM was higher than that in those without LNM (23.53% vs. 0.74%, *P* < 0.001). The details of the comparisons and *P*-values are shown in Table 1.

The univariate analysis of the clinicopathological factors assessed in the patients with and without LNM revealed a significant relationship between LNM and the tumor size (t = − 2.234, *P* = 0.026 < 0.05), pathology differentiation, and lymphovascular invasion (X^2^ = 23.593, 40.734, both *P* < 0.001). The LNM rates were higher in the patients with poorly differentiated carcinomas, tumors with a large diameter, and lymphovascular invasion. However, sex, age, tumor location, endoscopic morphology, depth of invasion and TB were not statistically significantly associated with LNM (*P* = 0.431, 0.316, 0.115, 0.703, 0.255 and 0.833, respectively; Table 1).

### Multivariate logistic regression analysis of the factors associated with LNM in ECC

A multivariate logistic regression analysis was used for the multivariate analysis of the following factors identified as significant in the univariate analysis: tumor size, pathology differentiation and lymphovascular invasion. The analysis showed that tumor size, pathology differentiation and lymphovascular invasion were risk factors for LNM in ECC (OR = 1.036 and *P* = 0.021; OR = 8.877 and *P* = 0.023; OR = 0.039 and *P* = 0.001; Table 2).

**Table 2:** Multivariate logistic regression analysis of ECC lymph node metastasis

Factors	OR	*P*-value	95% CI
**Tumor size**	1.036	0.021 *	1.005–1.068
**Pathology differentiation**	8.877	0.023 *	1.357–58.050
**Lympho-vascular invasion**	0.039	0.001 *	0.005–0.285

Note: * *P < 0.05*

### Kaplan–Meier estimates of overall survival and recurrence rates associated with chemotherapy in the no-LNM ECC patients

**Fig. 2:**
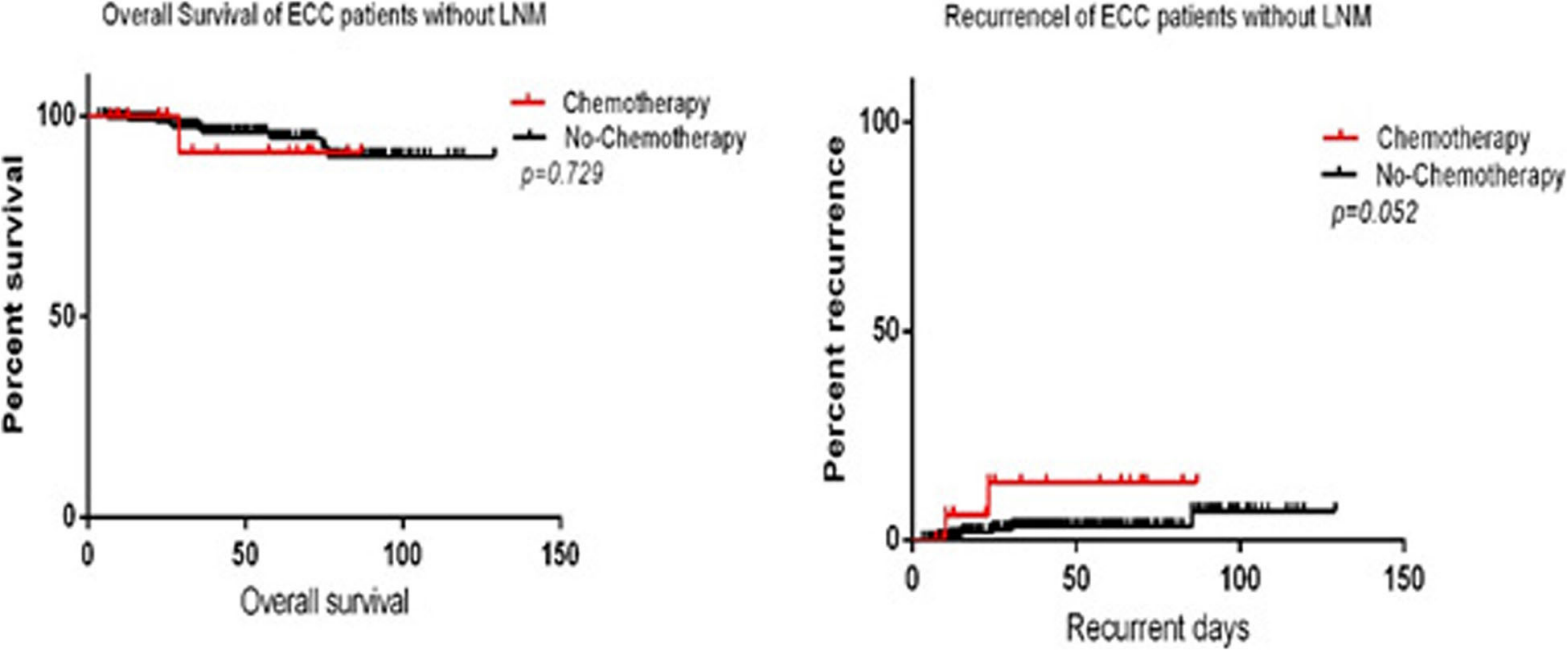
Overall survival and recurrence rate in followed-up ECC patients without LNM

The overall survival and recurrence rate in the no-LNM ECC patients was determined by a Kaplan–Meier analysis on prognostic differences between the chemotherapy (20 cases included) and no-chemotherapy (251 cases included) groups. The 11-year overall survival rates and recurrence rates were 95.94% (260/271) and 3.32% (9/271), respectively, in all 271 followed-up no-LNM ECC patients after surgery. Furthermore, 20 patients received chemotherapy after resection of the tumor, and 2 of these patients had recurrence, including 1 death. The main chemotherapy regimens included CapeOX (L-OHP + Cap) and FLOX (L-OHP + CF + 5-FU), and the periods of treatment ranged from 4 to 12 weeks. In this study, the patients treated with chemotherapy after surgery did not exhibit differences in the overall survival rates (95.0% vs. 96.02%, *P* = 0.729 > 0.05). Among the ECC patients without LNM, there were no significant differences between the chemotherapy and nonchemotherapy groups in overall survival and recurrence rates (Fig. 2).

### Kaplan–Meier estimates of overall survival and recurrence rates in LNM and no-LNM ECC patients matches according to baseline measures

**Fig. 3:**
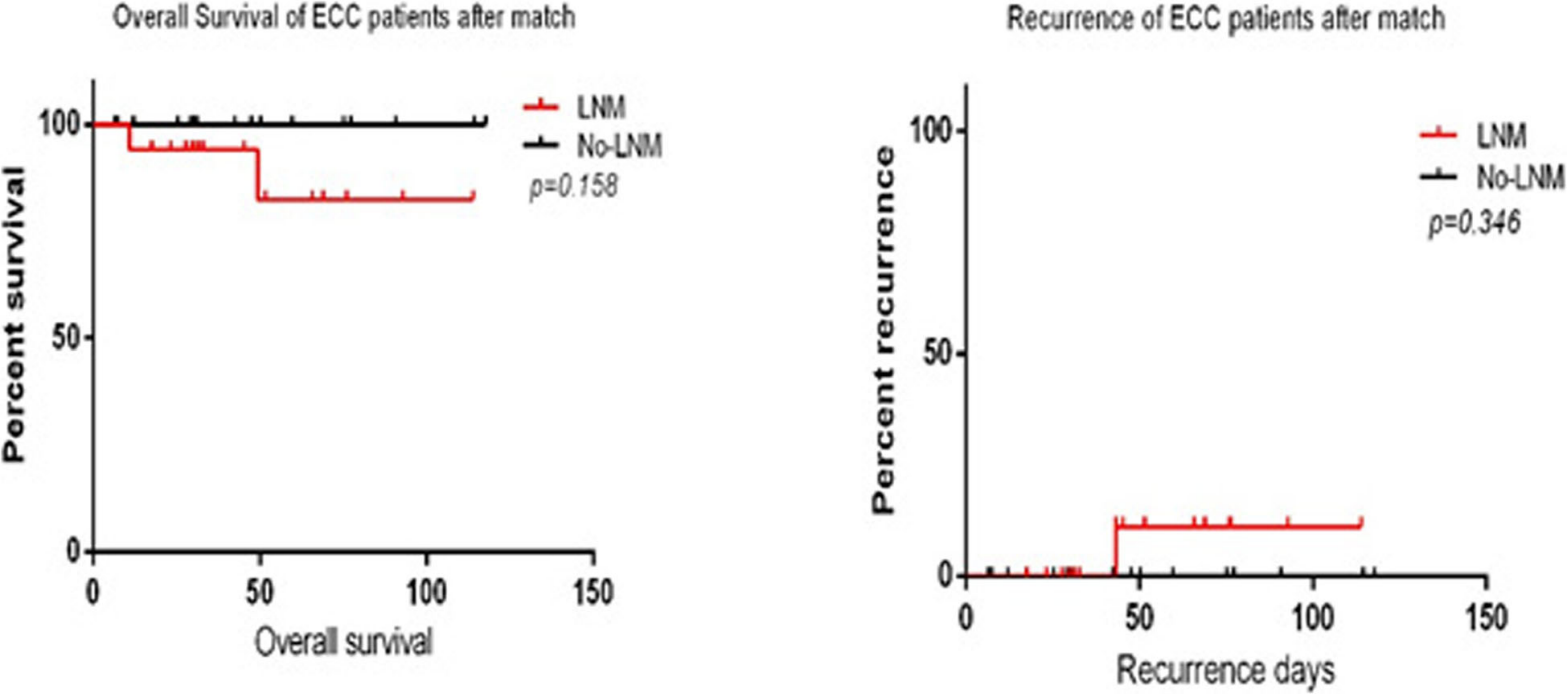
Overall survival and recurrence rate in followed-up ECC patients with and without LNM after matching

Seventeen ECC patients with LNM were matched with no-LNM patients. The baseline measure is shown in Table 3. However, more patients with LNM received chemotherapy (*P* = 0.034 < 0.05) and exhibited lymphovascular invasion (*P* = 0.033 < 0.05) than ECC patients with negative LNM. The Kaplan–Meier analysis showed that there were no significant differences in overall survival and recurrence rates between the ECC patients with LNM and without LNM (Fig. 3).

**Table 3:** Base-line of the ECC patients with and without LNM after matching

Factors	N *n* = 34	LNM(−) *n* = 17	LNM(+) *N =* 17	*P-value*
**Gender**				0.730
Male	15	7	8	
Female	19	10	9	
**Age (years old)**	57.1 ± 11.4	57.5 ± 12.3	56.8 ± 11.2	0.851
**Chemotherapy**				0.034*
Negative	27	16	11	
Positive	7	1	6	
**Smoking**				0.287
Negative	30	14	16	
Positive	4	3	1	
Alcohol				0.628
Negative	29	14	15	
Positive	5	3	2	
**Family history**				0.628
Negative	31	15	16	
Positive	3	2	1	
**Tumor size**	38.3 ± 17.3	38.3 ± 17.3	38.3 ± 17.3	1.000
**Tumor location**				1.000
Rectum	30	15	15	
Colon	4	2	2	
**Endoscopic morphology**				0.480
I (the uplift type)	30	14	16	
II (the flat type)	1	1	0	
III (the concave type)	3	2	1	
**Pathology**				0.504
Highly differentiated	3	2	1	
Medium differentiated	27	14	13	
Poorly differentiated	4	1	3	
**Depth of invasion**				1.000
Mucosal layer	4	2	2	
Submucosal layer	30	15	15	
Negative	30	17	13	
Positive	4	0	4	

Note: * *P < 0.05,* LNM(−):No lymph node metastasis, LNM(+):Lymph node metastasis

## Discussion

Early colorectal cancer is defined as an invasive adenocarcinoma of any size invading into, but not beyond, the submucosa with or without LNM. According to the 2000 cancer classification criteria of the WHO, when a tumor invades only the submucosa (pT1), it is defined as ECC. However, carcinoma in situ (Tis) and intramucosal carcinoma are customarily classified as ECC in China and Japan because their characteristics differ from those in Western countries. In total, 19 carcinomas in the epithelial layer, 57 carcinomas in the mucosal layer and 212 submucosal carcinoma cases were included in our study. In our study, the rate of LNM occurrence among the ECC cases was 5.90% (17/288). The rate of LNM has been previously reported to range from 7 to 15% [5–8], which is higher than our findings. Previously, it has been established that LNM may be highly correlated with lymphovascular invasion [10, 11], tumor size [12–17], TB [18], tumor invasion in the submucosa [19–23] and pathological differentiation [24–26]. In our study, lymphovascular invasion was identified as an independent risk factor for LNM in ECC. Moreover, the incidence of lymphovascular invasion in ECC patients with LNM was 23.53%, while that in the no-LNM patients was 0.74% (*P* < 0.001). In addition, the poorly differentiated cases accounted for 2.78% (8/288) of all cases. Our study confirmed that lymphovascular invasion, tumor size and pathological differentiation are risk factors for LNM in ECC patients by a multivariate logistic regression analysis. However, the pathological differentiation of the tumor is the most reliable predictor of LNM, which is supported by a meta-analysis of ECC [26]. As other studies have shown [27, 28], we found that the depth of tumor invasion in ECC patients was not related to LNM (*P* = 0.255 > 0.05). Some molecular alterations, such as P53, mitochondrial transcription factor A (TFAM), transforming growth factor (TGF)-β, adenomatosis polyposis coli (APC), mucin 2 (MUC2), hsa-miR-9-1, etc., might contribute to LNM in ECC patients [29–32]. Currently, whether chemotherapy is needed for ECC patients after tumor resection is still unclear. The National Comprehensive Cancer Network (NCCN) and Japanese Society for Cancer of the Colon and Rectum (JSCCR) guidelines recommend that local removal and regular follow-up are the standard treatments for selected ECC patients at the TisN0M0 and T1N0M0 stages [4, 31–34], while ECC patients with LNM are suggested to receive adjuvant chemotherapy after curative surgery. Among the ECC patients without LNM, chemotherapy did not seem to be beneficial for improving the overall survival and recurrence rates (Fig. 2).

However, Seyed Reza, et al. [35] found that the current guidelines for chemotherapy in T1N1M0 might not be necessary. Furthermore, we matched the LNM-ECC patients to other no-LNM patients according to the baseline measures (Table 3). More patients in the LNM group (64.71%) chose adjuvant chemotherapy than in the no-LNM group (5.88%, *P* = 0.034 < 0.05). In these two groups, chemotherapy was the only difference. Moreover, we found that there were no significant differences in overall survival and recurrence rates between the matched LNM and no-LNM groups (Fig. 3). Thus, our study finds that adjuvant chemotherapy could improve overall survival and reduce the recurrence rate in ECC patients with LNM. For advanced colorectal cancer, the treatments include systemic and targeted therapy (including immunotherapy). Targeted therapies consist of anti-tumor angiogenesis, anti-EGFR therapy, tumor immunoregulatory inhibitors, anti-BRAF therapy, and anti-HER-2 therapy [36].

In conclusion, this study shows that tumor size, pathological differentiation and lymphovascular invasion are the main risk factors for LNM in patients with ECC. The decision regarding whether to choose surgical or endoscopic resection should be made after considering the potential risks of LNM. However, the suggestion that ECC patients with LNM should receive adjuvant chemotherapy is still controversial. Our results verify that postoperative chemotherapy is necessary for ECC patients with LNM but might not be helpful for ECC patients without LNM. However, there are also some limitations in present study. First, a relatively small sample size was included in this retrospective study, and only 20 ECC patients without LNM received postoperative chemotherapy. Thus, it might be difficult to precisely estimate the association between postoperative chemotherapy and prognosis in LNM-negative ECC. Second, although both main chemotherapy regimens consist of a same key chemotherapeutic drug (Oxaliplatin), there may be still certain discrepancies in the curative effects between various regimens.

## Conclusion

In summary, tumor size, pathology, and lymphovascular invasion are risk factors for predicting LNM in ECC patients. We believe that it could be better and necessary for patients with ECC to undergo a full LNM evaluation before choosing surgical or endoscopic resection according to the risk factors of LNM, such as tumor size, pathology, and lymphovascular invasion. We confirmed that chemotherapy could improve overall survival and recurrence in ECC patients with LNM after resection, while it may be unnecessary for ECC patients without LNM to receive chemotherapy after tumor resection. Considering these above limitations, this result should be interpreted with caution, and further well-designed, prospective, large sample study is imperative to verify the association between postoperative chemotherapy and prognosis in ECC patients without LNM.

## Data Availability

All analyzed data are included in this published article. The original data are available upon reasonable request to the corresponding author.

## Abbreviations

LNM: Lymph node metastasis
ECC: Early colorectal cancer
TB: Tumor budding
NCCN: National Comprehensive Cancer Network
L-OHP: Oxaliplatin
CAP: Capecitabine
CF: Calcium folinate
5-FU: 5-fluorouracil
Chemo: Chemotherapy
TFAM: Mitochondrial transcription factor A
TGF-β: Transforming growth factor-β
APC: Adenomatosis polyposis coli
